# M. Ricord on the Treatment of Gonorrhœal Ophthalmia

**Published:** 1842-04-30

**Authors:** 


*On the Treatment of Gonorrhœal Ophthalmia.* By M. Ricord.—From the dangerous nature of this affection, the surgeon should be careful to attack it on the earliest appearance of any symptoms which indicate its development. As soon as the conjunctiva shows the least redness, whether general or partial, the eye should be protected from the light, strict attention to regimen ordained, and the bowels opened by some laxative medicine. When the gonorrhœa is accompanied by some fever, or the latter appears immediately before or soon after the redness of the eyes, leeches are to be applied on the temple. The main remedy, however, is cauterisation of the affected conjunctival membrane with the nitrate of silver. It is far better to run the chance of employing this measure, in cases of doubtful appearance, than to defer it until the disease has acquired a degree of intensity which may baffle all our efforts. In a very early stage, one or two light applications of the caustic may be sufficient; but in all cases the subsequent use of the remedy must be regulated by the effect of the first applications on the inflammation of the eye. The direct use of the nitrate of silver is seconded by a wash containing the same substance in solution. One grain to the ounce of distilled water is commonly sufficient, and the lotion may be applied three or four times a day. In more severe cases, when the conjunctiva is granular, it furnishes an abundant secretion, and the eyelids are more or less swollen, but when no regular chemosis exists, we still rely on the nitrate. Care must be taken to apply it to all the affected parts, but not to touch the cornea. The effects of this remedy, when prudently but energetically used, are truly wonderful; there are certain rules, however, by which the practitioner should be guided. When the first application fails to diminish the tumefaction, pain, and secretion of pus, and especially when the appearance and consistence of the latter are not changed by it, we must have recourse to the nitrate of silver again; in very acute cases this must be done within five or six hours of the same day.

The nitrate of silver has been applied in the form of solution, as a powder, or solid; but the lotion is certainly the most convenient form, particularly in the case of children or irritable persons. The strength in which I employ the solution is two scruples of the nitrate to eight scruples of water. It may be applied with a camel's hair brush, first to the lower lid, then to the upper one, and finally to the globe of the eye. I have already said that we should be careful to avoid the cornea. Some recommend the application of a drop of oil to cover this part of the eye; but the object is equally well attained by injecting some water between the eyelids. Solid nitrate of silver is the form which I generally prefer; with the caustic pencil the granulations and other morbid products are more easily commanded, but more caution is required to avoid injuring the cornea. The pencil should be passed over the diseased surfaces, until a thin white film ensues; our object being to modify the action of the mucous membrane, but not to destroy it.

Although I have thus recommended the use of nitrate of silver in all stages of gonorrhœal ophthalmia, I would not have the practitioner neglect other means of subduing the inflammation. In severe cases, general and local abstraction of blood must be employed; we must command the inflammation, and not be content to follow it; purgatives and abstinence from food will also be requisite. The patient, likewise, finds much relief from warm fomentations of the decoction of poppies. The extract of belladonna is efficacious in all cases of ophthalmia, and in this form particularly, by diminishing the sensibility of the organ; it may be rubbed round the base of the orbit twice a day. To cleanse the eye from the purulent secretion with which it is constantly bathed, is also indispensable; for this purpose I am in the habit of injecting, every hour or half hour, according to circumstances, the anodyne decoction and weak lotion of lunar caustic alternatively. The efficacy of the treatment now recommended has been abundantly proved by experience. During the last ten years I have only lost a single eye, and this can be testified by the numerous practitioners and pupils who have attended the venereal hospital during that time.—*Prov. Journ., from Bul. de Therapeutique.* Jan. 1842.

